# Synthesized Attributes of Water Use by Regional Vegetation: A Key to Cognition of “Water Pump” Viewpoint

**DOI:** 10.1155/2014/954849

**Published:** 2014-07-24

**Authors:** Xin-hui Huang, Fu-ke Yu, Xiao-ying Li, Yuan Zheng, Hua Yuan, Jian-gang Ma, Yan-xia Wang, Dan-hui Qi, Hong-bo Shao

**Affiliations:** ^1^College of Environmental Science and Engineering, Southwest Forestry University, Kunming 650224, China; ^2^Institute of Environmental Sciences and Ecological Restoration, College of Ecology and Environmental Sciences, Yunnan University, Kunming 650091, China; ^3^Key Laboratory of Coastal Biology & Bioresources Utilization, Yantai Institute of Coastal Zone Research (YIC), Chinese Academy of Sciences (CAS), Yantai 264003, China

## Abstract

Recently, the frequent seasonal drought in Southwest China has brought considerable concerns and continuous heated arguments on the “water pump” viewpoint (i.e., the water demand from *Hevea* spp. and *Eucalyptus* spp. can be treated as a water pump) once again. However, such viewpoint just focused on water consumption from vegetation transpiration and its ecoenvironment impacts, which had not considered other attributes of vegetation, namely, water saving and drought resistance, and hydrological regulation (water conservation) into consideration. Thus, in this paper, the synthesized attributes of regional vegetation water use had been mainly discussed. The results showed that the study on such aspects as the characters of water consumption from vegetation transpiration, the potential of water saving and drought resistance, and the effects of hydrological regulation in Southwest China lagged far behind, let alone the report on synthesized attributes of water utilization with the organic combination of the three aspects above or the paralleled analysis. Accordingly, in this paper, the study on the synthesized attributes of water use by regional vegetation in Southwest China was suggested, and the objectives of such a special study were clarified, targeting the following aspects: (i) characters of water consumption from transpiration of regional typical artificial vegetation; (ii) potential of water saving and drought resistance of regional typical artificial vegetation; (iii) effects of hydrological regulation of regional typical artificial vegetation; (iv) synthesized attributes of water use by regional typical artificial vegetation. It is expected to provide a new idea for the scientific assessment on the regional vegetation ecoenvironment effects and theoretical guidance for the regional vegetation reconstruction and ecological restoration.

## 1. Introduction

In Southwest China, a global hot place for its abundant biodiversity and a place with frequent seasonal drought, an unprecedented transseasonal constant severe drought took place in 2010, which aroused considerable concerns in China and even worldwide [1, 2]. Historically speaking, though several droughts broke in this area [3], the drought in 2010 was the severest with many socioeconomic losses and far-reaching impacts [3, 4]; in addition, since 2010, droughts continuously took place in the following consecutive 4 years in such areas including Kunming, which had brought about public concerns at all social levels [5–7]. Accordingly, there were many scientific issues worth discussing behind the drought, among which the relationship between regional drought and regional artificial vegetation has become a scientific issue with rather considerable concerns recently [8].

In the past several decades, a great deal of artificial vegetation has been established targeting regional economic development and ecoenvironment protection in each province (autonomous region, municipality) in Southwest China. For example, the area of the land used for the plantation of* Eucalyptus* spp. in Guangxi, Yunnan, Guizhou, and the plantation of* Hevea* spp. in Yunnan have been considerably large. However, quite a few ecoenvironment problems have been brought by such artificial vegetation, such as the worsening of soil water and nutrition for the land [9, 10] and functional degradation of the ecosystem [11, 12] and decrease in biodiversity and land productivity [13, 14]. The “water pump” viewpoint of* H.* spp. (*E.* spp.) prevailing in the past and the description of the cause of severe droughts in Southwest China as “a human disaster” prevailing in recent years were just in accordance with such ecoenvironment problems.

Undoubtedly, those in favor of “water pump” viewpoint did analyze ecoenvironment effects of regional typical artificial vegetation such as* H.* spp. and* E.* spp. from the standpoint of water consumption. However, the water ecological relationship of vegetation was not just limited to water consumption from transpiration; such attributes as water saving and drought resistance and hydrological regulation (water conservation) should also be included. Clearly, just to explore the ecoenvironment impacts of artificial vegetation from the perspective of water consumption was far from enough. Accordingly, it is suggested in this paper that only through the systematical analysis on the synthesized attributes of vegetation, including water consumption (transpiration), water saving (drought resistance), and water conservation (hydrological regulation), can the ecoenvironment effects of artificial vegetation be mastered comprehensively and further to objectively assess the relation between regional vegetation succession and regional drought.

Consequently, synthesized attributes of regional vegetation water use were mainly discussed in this paper, according to such research background above. Based on the all-round analysis on the status quo and development trends for characters of water consumption from vegetation transpiration, their potential of water saving and drought resistance, and their effects of hydrological regulation, a special study on the synthesized attributes of water use of typical artificial vegetation in Southwest China was suggested in this paper, in order to provide a new idea for the objective, scientific, and all-round assessment on the ecoenvironment effects of regional vegetation and further to provide a theoretical guidance for the reconstruction and recovery of regional vegetation, as well as a scientific cognition of the relationship between regional drought and regional artificial vegetation.

## 2. Study Status Quo and Development Trends

As mentioned above, vegetation water use includes three attributes: water consumption (transpiration), water saving (drought resistance), and water conservation (hydrological regulation), respectively, representing the direct water consumption from vegetation transpiration, vegetation self-adjustment to extreme moisture condition—drought stress, and its maintenance and regulation of such ecological hydrological conditions as rainfall, runoff, and soil water. The study status quo and development trends related to the topic in this paper would be illustrated in such three aspects above.

### 2.1. Water Consumption from Vegetation Transpiration

The character of water consumption from plant transpiration is one of the core ingredients in the study of plants' water physiology and ecology. Since the 21st century, quite a few scholars have conducted studies on water consumption from vegetation transpiration targeting regional vegetation construction and ecological restoration (Table 1). For example, Sui et al. [15] measured the daily transpiration rate of such commonly seen species in Loess Plateau regions as* Forsythia suspense*,* Lonicera japonica*,* Rubus crataegifolius*, and* Swida alba*. Xu et al. [16] discovered the laws of water consumption from vegetation transpiration of* Haloxylon ammodendron* in the hinterland of Taklimakan desert and the influential environment factors of stem sap flow. Wang et al. [17] analyzed the characteristics of daily, monthly, and seasonal changes in water consumption from transpiration of* Magnolia liliiflora* as a green species in Beijing City. Wang et al. [18] discovered the relationship between water consumption from transpiration and soil water of flue-cured tobacco species. Zhang et al. [19] studied the regulatory role of surface sand-covering in water consumption from transpiration of apple trees, and the list can go on [20–25, 40]. Such findings illustrated that the study on water consumption from transpiration shifted to such synthesized studies as the analysis of its environmental influential factors, the development of large-scale research methods (e.g., scale transformation, model calculation, etc.), and the application of manmade regulation technologies (e.g., surface sand-covering, etc.) from the simple determination of transpiration rate (strength) of a single species, and the experimental species covered regional unique species for ecological recovery, landscape greening species, economic crops, horticultural plants, and so forth, and the study scale also extended to ecological system (e.g., forest stands) from individuals. However, through the comparative analysis of current literature, it is found that the study on water consumption from transpiration was still focused on species in arid or semiarid region, and the study on plants in other climatic province was rather weak. For example, as for the severe drought-stricken regions in Southwest China in 2010, the study on the characters of water consumption from transpiration of ecological recovery tree species in dry-hot river valley in Jinsha River [26] was the only report on such topic. Thus, such study status quo showed the double bottle-neck of deficiency in the basic theory and lack of technological methods that will hinder vegetation recovery and reconstruction in Southwest China and the prevention and treatment of such disaster as drought. Obviously, it is rather urgent to shift the study focus on water consumption from regional vegetation transpiration to the regions with uneven water resources and frequently drought-stricken areas.

### 2.2. Potential of Water Saving and Drought Resistance of Plants

The study on water saving and drought resistance of plants plays a significant scientific role in the agricultural and forestry development in arid and semiarid areas. In recent several years, the study on it at home and abroad gained a rapid growth in the following three aspects (Table 2): (i) the formation mechanisms of drought resistance and its influential factors. The recent related research results showed that there were many mechanisms, including ecological, morphological, anatomical, and physiological, and so forth, for the formation of drought resistance [27], and its influential factors included morphological adaptability characters, photosynthetic physiological index, the activity of oxidase, malondialdehyde (MDA) concentration, permeation regulation, hormone and stoma regulation, protein induced by drought, and stable carbon isotope[27]; (ii) the determination and assessment of drought-resisting capacities and the selection of drought-resisting species as well. Because of the complexity in the formation of drought-resisting mechanism and its influential factors, a single index could not meet the demand of scientific determination and comprehensive assessment of plants' drought-resisting capacities. Quite a few scholars had actively explored the assessment system and comprehensive assessment methods of multiple indexes for drought resistance of plants, and some rather good results had gained on such aspects as the selection of identification index for drought-resisting of some crops, the construction of comprehensive assessment system, the classification of drought-resisting capability of species, and the exploitation of drought-resisting species [28–31]. Accordingly, in some countries and regions, some regulations on drought-resisting identification and assessment were established for some main crops such as wheat, corn, and beans; (iii) the basis of artificial improvement and regulation for drought resistance. Presently, it is discovered that such methods as elimination of active oxygen inside the plants [32], accumulation of antioxidant substance [33], gene expression of drought response [34, 35], AM fungus inoculation [36], allogenic material implementation [37, 38], and drought hardening [39] can boost plants' drought resistance; such basic researches would provide strong scientific supports for R & D of technologies used in artificial improvement and regulation of plants drought resistance. However, there were also some problems existing in the study of plants' drought resistance. For example, the study objects were focused mainly on major crops, horticultural plants, and grassland species in arid and semiarid areas, while the study on forest vegetation, especially ecological recovery trees and artificial economic trees, was far from abundant. Such problems had brought direct impacts on and potential threats to the regional forestry development and ecological recovery, which should be solved as soon and as early as possible.

It must be pointed out that the researches on drought-resistance mechanism of plants at molecular and cellular level sprang up consecutively in recent years.

Studies showed that when under the water stress, plants would make a positive response [41–46] via the transfer and transduction of intercellular and intracellular adverse signal [47–50], which indicated the rapid perception and positive adaptation of plants towards environmental changes. Such process includes the following 3 links: (i) perception of cells or organizations to water stress and production of intercellular messengers afterwards; (ii) intercellular messengers transfer between cells or organizations and eventually arrive at the acting locus of the recipient cells; (iii) recognition, reception, transduction, and gene expression of recipient cells towards intercellular messengers, eventually leading to the physiological and functional optimization in cells; thus plants are characterized with the adaptation or resistance towards the stress [51].

#### 2.2.1. Perception and Recognition to Water Stress Signals

There are diverse stress signals which can be perceived by cells or organizations [41, 43, 46, 52], including mechanical stress signals (plasmolysis of plants cells resulting from drought stress), osmotic stress signals (changes in transmembrane osmotic potential of plant cells resulting from water deficit), oxidative stress signals (production of free radical such as H_2_O_2_ *·*,  H_2_O*·* and active oxygen in plant chloroplast resulting from water stress), and defensive stress signals (changes in endogenous hormones of plant such as abscisic acid, ethylene, and polyamine resulting from drought stress); furthermore, the way such signals above are perceived and recognized is also characterized by diversity [41, 43, 46, 52].

#### 2.2.2. Intracellular Transduction of Water Stress Signals

Intracellular transduction is a step-by-step process of signal transferring and amplifying via protein phosphorylation and dephosphorylation in plant. In this process, protein kinase is a key component. It has been found that quite a few protein kinases participated in the signals transduction of drought, mainly including CDPK (calcium-dependent protein kinase) [53] and MAPK (mitogen-activated protein kinase) [54].

#### 2.2.3. Gene Expression Induced by Water Stress Signals

**Figure 1:**
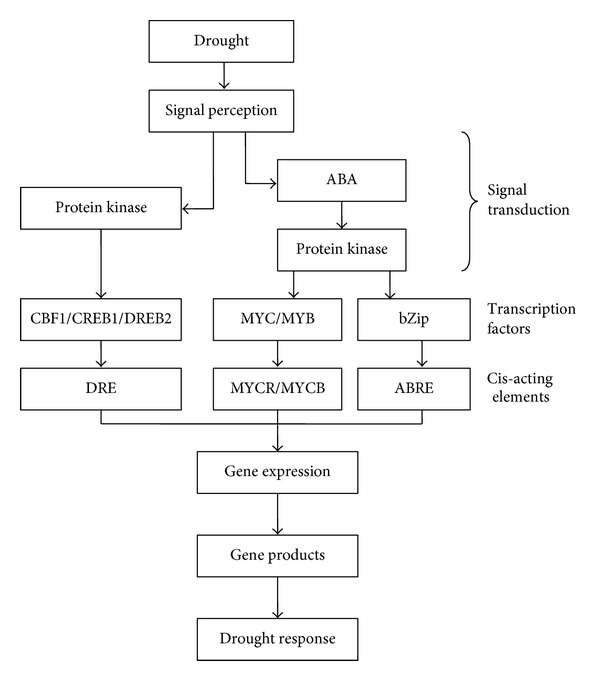
Signal transduction and gene expression in plant response to drought stress.

The perception and transduction to water stress signals activate a series of protein kinases, eventually leading to increase in concentration and activity of related transcription factors and the combination with relevant cis-acting elements, to further induce gene expression. Such process falls into two pathways—ABA dependent pathway and ABA independent pathway [50, 55, 56] (Figure 1).

Many plant genes can be induced to express via drought [57]. The genes induced to express can be divided into two categories according to functional ways—regulatory protein gene and functional protein gene [55]. The products coded by the former play a regulatory role in signal transduction and gene expression, such as transcription factors, and the products coded by the latter play a direct protecting role in the process of plant drought-resistance, mainly including key enzymes to compound osmotic regulatory materials, enzymes to eliminate active oxygen, and protein to protect biomacromolecule and cytomembrane structure [45].

### 2.3. Hydrological Regulation Effects of Vegetation

Hydrological regulation effects of vegetation play an irreplaceable role in maintaining regional ecological security and environment health. Recently, some rather influential achievements have been obtained in the scientific study of such field (Table 3). For example, in the study on Loess area, it is found that the change in land use pattern and vegetation spatial pattern would lead to differences in the runoff and sediment yield [58–60]; the analysis on the* Picea crassifolia* forest in Qilian Mountain, Qinghai, illustrated that the ecological hydrological indexes of forest stands such as total penetrated rainfalls, interception, and trunk stream were greatly influenced by canopy structural characteristics [61]; the study on the reservoir area of Three Gorges showed that the general ecological hydrological function of the forest ecosystem in the reservoir area was represented as shrub forest > mixed conifer-broadleaf forest, evergreen broad-leaved forest >* Phyllostachys pubescens* forest > cropland, among which the ecological hydrological function of forest vegetation was 1.12~2.90 times of that of cropland, and the ecological hydrological function of shrub forest was the best [62], and the list could go on [63]. There were few reports on the ecological hydrological regulation effects of vegetation in Southwest China 10 years before [64, 65]; several years afterwards, there was almost no report, though only in recent years the study on this issue got warm again after a cold spell [66–70]. However, from the general development status quo in such field at home and abroad, the study on the hydrological regulation effects of vegetation was characterized with regional imbalance; namely, it was mostly focused on vegetation in arid or semiarid area, while the study on vegetation in other climatic regions was rather deficient. Take Southwest China for example, there were few research projects approved on such theme recently and before, in addition to lack of link between research topics selected as well as achievements in series. Considering it from the perspective of regional environment health and sustainable development, it is rather urgent to strengthen the study in this field.

From the analysis above, the scientific researches on water consumption from vegetation transpiration, water saving and drought resistance, and hydrological regulation effects have gained great achievements or a rapid growth recently. However, there was a regional imbalanced study in each field, especially in Southwest China, a place frequently stricken with seasonal droughts; the study in each field mentioned above developed slowly and lagged far behind. Furthermore, there was no report on the synthesized attributes of water use by vegetation from the standpoint of the comprehensive assessment based on the study with the paralleled analysis or organic combination of the three. Such study status quo has become a major barrier for the regional vegetation construction and ecological recovery as well as the development and utilization of related disciplines. Consequently, it is of great urgency to launch and strengthen the scientific study on such related disciplines.

## 3. Research Suggestions and Major Targets

Based on the research status quo and development trends at present, together with the strategic consideration and technical demands for the comprehensive analysis on the ecoenvironment effects of vegetation and scientific recognition of the cause of regional drought, it is suggested in this paper that a special project should be launched from the perspectives of water consumption from transpiration, water saving and drought resistance, and water conservation to conduct a special research on synthesized attributes of water use by regional vegetation. Such project meets the strategic goal and technological demands of national and regional ecoenvironment protection, which was expected to provide scientific evidences and theoretic supports for the regional vegetation construction and ecological restoration together with the scientific cognition of the relationship between regional drought and regional artificial vegetation. Thus the major targets and key research contents are as follows.

**Figure 2:**
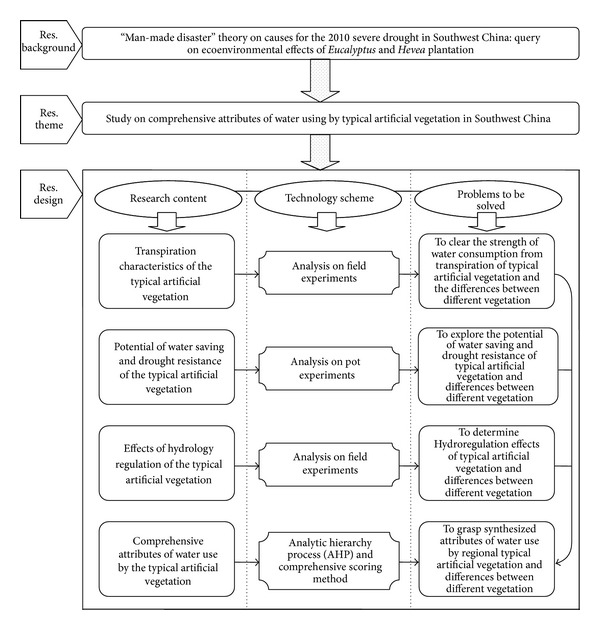
Technical route for synthesized research on water use by regional vegetation.

*(i) Characteristics of Water Consumption from Transpiration by Regional Typical Artificial Vegetation.* Based on the selection of research regions and typical artificial vegetation, together with field experiments, such characters of regional typical vegetation as stem sap flow density, water consumption from transpiration, characters of daily and seasonal change, and influential environment factors were studied, to reveal the water consumption strength of typical artificial vegetation as well as its temporal-spatial characters and further to clear the interspecies differences of different vegetation species in water consumption from transpiration (Figure 2).

*(ii) Potential of Water Saving and Drought Resistance of Regional Typical Artificial Vegetation.* A test study flat should be established with pot experiments as the dominance, to study the characters of morphological and physiological changes of typical artificial vegetation species under the stress of drought and to explore its response mechanism and adaptability strategy towards the stress of drought, and further to clear the potential of water saving and drought resistance of typical artificial vegetation and the interspecies differences in such attributes based on the selection of identification index of drought-resisting capacity and the establishment of assessment system for drought-resisting capacity (Figure 2).

*(iii) Hydrological Regulation Effects of Regional Typical Artificial Vegetation.* On the basis of experiment study system with the observation of field runoff plots, hydrological regulation effects and water conservation functions of typical artificial vegetation in different regions, such as interception of atmospheric precipitation, the conservation of soil water, and the decrease in runoff and soil erosion together with sediment transfer, should be studied, to clear the role of regional typical vegetation in hydrological regulation and water conservation and the differences between different vegetation as well (Figure 2).

*(iv) Synthesized Attributes of Water Use by Regional Typical Artificial Vegetation.* Targeting regional typical artificial vegetation, a structure model of the synthesized attributes of water use should be established, together with the introduction of synthesized assessment methods, to systematically analyze the synthesized attributes of typical artificial vegetation in such three aspects as water consumption from transpiration, water saving and drought resistance, and hydrological regulation, to further reveal the total strength of impacts of regional typical artificial vegetation on ecoenvironment through water use and the differences in such strength between different vegetation in a comprehensive way (Figure 2).

## Figures and Tables

**Table 1:** Typical cases of study on transpiration characteristics of plant species.

Study area	Subject investigated	Study method	Literature source
Loess Plateau, China	*Forsythia suspense*, *Lonicera japonica*, *Rubuscrataegifolius*, and *Swida alba *	Rapid weighing method	Sui et al. (2010) [15]

Hinterland of Taklimakan desert, China	*Haloxylonammodendron *	Heat-balance technique	Xu et al. (2008) [16]

Beijing City, China	*Magnolia liliiflora *	Thermal dissipation method	Wang et al. (2011) [17]

Wuhan, China	Flue-cured tobacco	Artificial water control experiment in the shed of rain-free condition	Wang et al. (2007) [18]

Pingliang of Gansu Province, China	Apple trees	Thermal diffusion method	Zhang et al. (2010) [19]

Hinterland of Taklimakan desert, China	*Calligonumarborescens *	By using heat-balance stem flow gauge	Shan et al. (2012) [20]

Urumqi of Xinjiang, China	*Pinaceaeschrenkiana* stands	Thermal diffusion method and Fitting function method	Zhang et al. (2011) [21]

Miyazaki and Akita, Japan	Forest vegetation	Model analysis method	Komatsu et al. (2008) [22]

Northwest China	*Caraganaintermedia*, *Hippophaerhamnoides*, *Hedysarum leave*, and *Salix psammophila *	By using porometer, leaf area meter, and Mettler electronic balance combined with calculation	Tong et al. (2008) [23]

Southeastern Arizona, USA	*Eragrostislehmanniana*, *Heteropogoncontortus *	A chamber-based isotope method	Yepez et al. (2005) [24]

Central Sweden, Eastern China, and Northwestern Cyprus	Different climatic regions	Model analysis method	Xu and Singh (2005) [25]

Dry-hot valleys of the Jinsha River in Yunnan, China	28 potted afforestation species (e.g., *Ziziphusmauritiana*, etc.) for vegetation restoration	Rapid weighing method	Duan et al. (2009) [26]

**Table 2:** Typical cases of study on potential of water saving and drought-resistance of plant species.

Research topic	Subject investigated	Study method	Literature source
Drought resistance (tolerance) mechanism	Plant species in arid area of China	Comprehensive review method	Li et al. (2010) [27]

Drought resistance identification and evaluation	Backcross lines of Dongxiang common wild rice (*Oryzarufipogon*Griff.)	Correlation analysis combined with stepwise regression analysis	Fu et al. (2012) [28]

Drought resistance identification and evaluation	Different peanut varieties widely grown in Northern China	Drought coefficient method and Subordinate function value method	Zhang et al. (2012) [29]

Drought resistance identification and evaluation	*Brassica napus*L.	Principal component analysis, regression analysis, and clustering analysis	Zhu et al. (2011) [30]

Drought resistance identification and evaluation	Nine marigold cultivars	Principal component analysis, subordinate function, and cluster analysis	Tian et al. (2011) [31]

Basis for drought resistance regulation (improvement)	Crop plants	Comprehensive review method	Gill and Tuteja (2010) [32]

Basis for drought resistance regulation (improvement)	Bread wheat (*Triticumaestivum* L.).	Correlation analysis method	Osipova et al. (2011) [33]

Basis for drought resistance regulation (improvement)	*Arabidopsis* spp.	Vacuolar localization method	Wang et al. (2011) [34]

Basis for drought resistance regulation (improvement)	Transgenic tobacco (*Nicotianatabacum* cv. *Xanthi*) plants	Technologies method of molecular biology	Faize et al. (2011) [35]

Basis for drought resistance regulation (improvement)	*Trifoliumalexandrinum *	By using molecular biology technologies	Zézé et al. (2008) [36]

Basis for drought resistance regulation (improvement)	*Haloxylonammodendron *	Completely randomized plot experiment	Kang et al. (2012) [37]

Basis for drought resistance regulation (improvement)	*Arabidopsis thaliana *	Isotopic tracer method	Ikegami et al. (2009) [38]

Basis for drought resistance regulation (improvement)	Two psammophytes (*Setariaviridis* and *Digitariaciliaris*)	Completely randomized plot experiment	Luo et al. (2011) [39]

**Table 3:** Typical cases of study on hydrological regulation effect of vegetation.

Study area	Subject investigated	Study method	Literature source
Loess Plateau, China	Slope with different land use pattern	Field plot experiment	She et al. (2011) [58]

Loess Plateau, China	Five different land use types	Field plot experiment	She et al. (2010) [59]

Menglun, Xishuangbanna, Southwest China	Ten land use categories in the study area	By using RS and GIS technology	Hu et al. (2009) [60]

the Qilian Mountains, China	Qinghai spruce (*Piceacrassifolia*) forest	Field observation experiment	Tian et al. (2012) [61]

Three Gorges Reservoir area, China	5 typical vegetation types (mixed conifer and broadleaf forest, evergreen broadleaved forest, etc.)	Analytic hierarchy process (AHP) and comprehensive scoring method	Y. Q. Wang and Y. J. Wang (2010) [62]

—	Forest ecosystems	Comprehensive review method	Neary et al. (2009) [63]

Xishuangbanna, Southwest China	Tropical seasonal rain forest and rubber forest	Water balance method	Zhang et al. (2003) [64]

Central Yunnan, China	*Pinusyunnanesis* plantation, *Eucalyptus maidenii* plantation, and their mixed plantation	Field observation combined with sampling and analysis	Wang et al. (2001) [65]

Ailao Mountain, Yunnan, China	Evergreen broadleaf forest	Field observation combined with sampling and analysis	Qi et al. (2012) [66]

Xishuangbanna, Southwest China	Native tropical rain forest and artificial rubber plantation	Isotopic tracer method	Liu et al. (2011) [67]

Central Yunnan, China	*Eucalyptus* plantation, *Pinus* plantation, shrubland, and seminatural and natural secondary forests.	Field observation combined with sampling and analysis	Hou et al. (2010) [68]

Xishuangbanna, Southwest China	Tropical rain forest and rubber plantation	Isotopic tracer method	Liu et al. (2008) [69]
